# Case report: Blepharospasm in peak-dose dyskinesia may benefit from amantadine in Parkinson's disease

**DOI:** 10.3389/fneur.2022.961758

**Published:** 2022-09-30

**Authors:** Qian-Ya Fan, Xiao-Dong Zhang, Ze-Di Hu, Shi-Shi Huang, Shi-Guo Zhu, Cai-Ping Chen, Xiong Zhang, Jian-Yong Wang

**Affiliations:** ^1^Department of Neurology, The First People's Hospital of Jiande, Hangzhou, China; ^2^Department of Cerebral Surgery, The First People's Hospital of Jiande, Hangzhou, China; ^3^Institute of Geriatric Neurology, Department of Neurology, The Second Affiliated Hospital and Yuying Children's Hospital, Wenzhou Medical University, Wenzhou, China; ^4^Central Blood Bank of Jiande, Hangzhou, China

**Keywords:** blepharospasm, Parkinson's disease, peak-dose dyskinesia, amantadine, dyskinesia

## Abstract

**Introduction:**

Blepharospasm is uncommon in Parkinson's disease, especially in the peak-dose dyskinesia period.

**Case presentation:**

We herein present the case of a patient with PD who developed blepharospasm in the peak-dose dyskinesia period. The symptom was improved by taking amantadine.

**Conclusion:**

The current report expands the phenomenology of peak-dose dykinesia in PD to include dystonic blepharospasm. This complication of levodopa therapy may respond to amantadine despite the dystonic appearance of movements.

## Introduction

Parkinson's disease (PD) is a common age-related neurodegenerative disorder, with bradykinesia, rest tremor, and rigidity as its core features (1). In a more advanced stage, management of motor fluctuations, drug-resistant symptoms, and non-motor features become challenges and can reduce the quality of life (2, 3).

Peak-dose dyskinesia is the most common type of levodopa-induced dyskinesia, and it occurs during the plateau of levodopa plasma levels. Peak-dose dyskinesia usually consists of chorea, dystonia, and, less commonly, myoclonus in the head, trunk, and limbs (4–6). Sometimes, eye-related involuntary movements may be present in peak-dose dyskinesia and are usually accompanied by dyskinesia in other parts of the body. Conjugate involuntary upward or lateral eye deviation is uncommon but has been described (7–10).

Apraxia of eyelid opening (AEO) and blepharospasm in patients with PD are often of concern because they obstruct the patients' visual field. AEO is characterized by non-paralytic inability to reopen the eyes without a spasm of the orbicularis oculi muscle, while blepharospasm is an involuntary spasm of the orbicularis oculi muscle and is considered focal dystonia (11). It is more prevalent in atypical parkinsonism, especially progressive supranuclear palsy (PSP) (12, 13). However, in patients with idiopathic PD, blepharospasm is more likely to occur during “off” periods and is usually accompanied by dystonia in other parts of the body (4).

Herein, we report a female patient with PD who developed blepharospasm as the main manifestation of peak-dose dyskinesia. The symptom was relieved by taking amantadine.

## Case presentation

**Figure 1:**
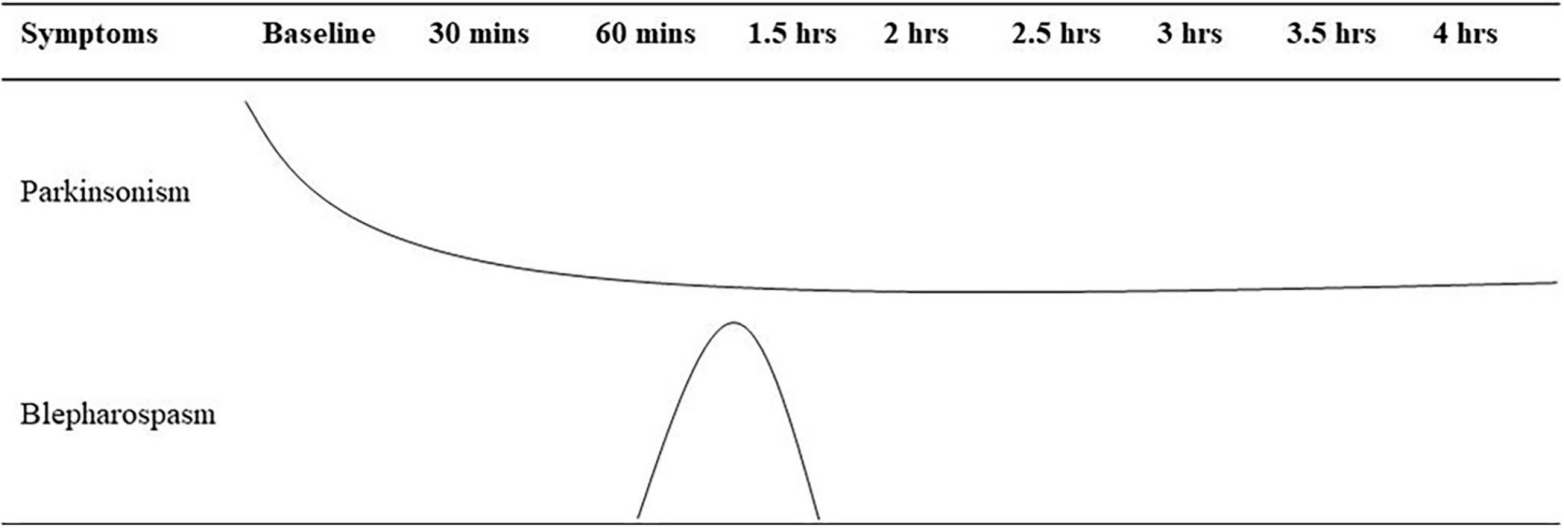
Summary of the levodopa challenge test for the case. Mins, minutes; hrs, hours.

**Figure 2:**
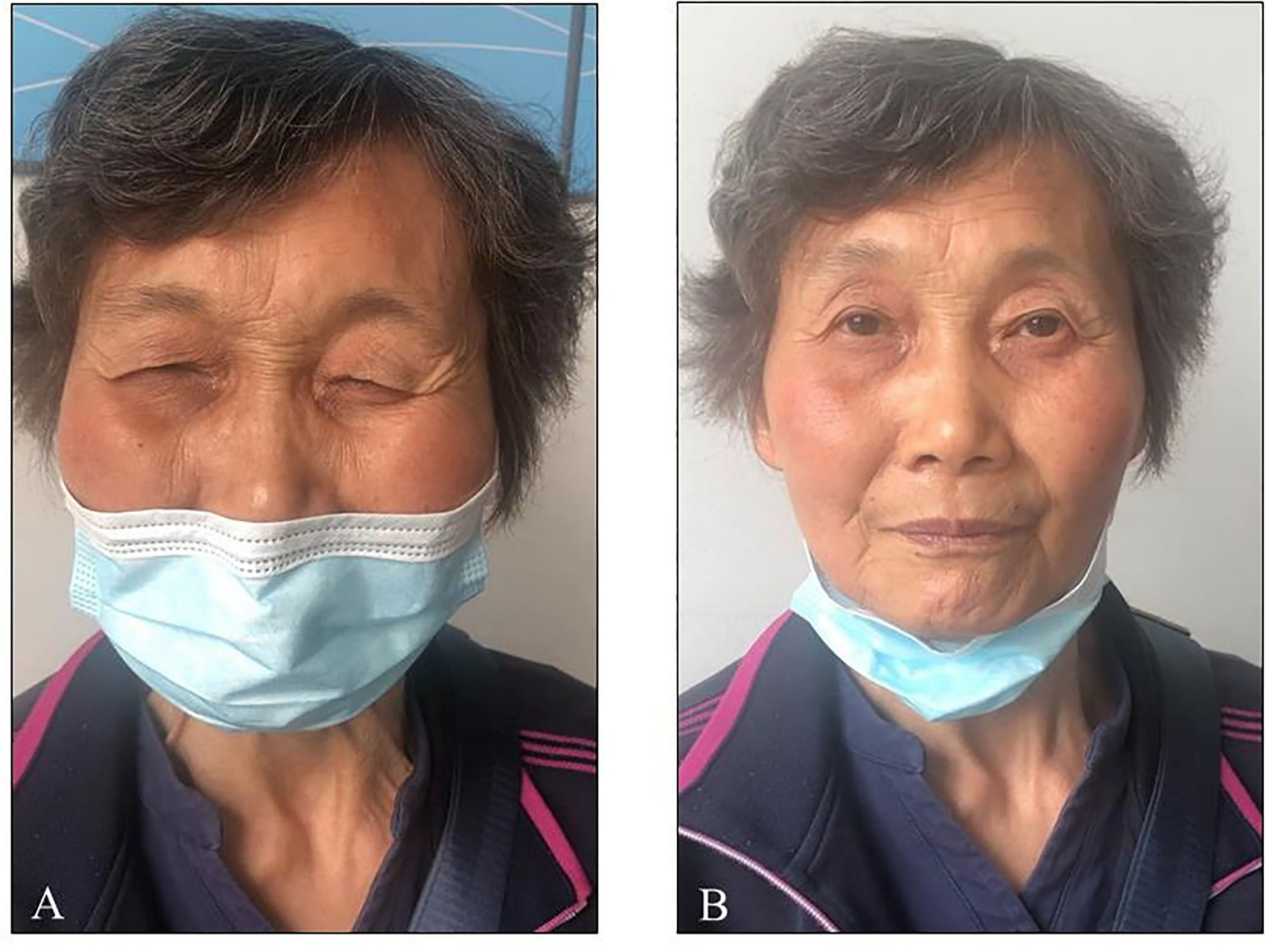
**(A)** Patient took madopar for 1 h. **(B)** Patient took madopar for 1.5 h.

The patient is a 71-year-old Chinese woman who has been displaying bradykinesia, resting tremor in her left limbs, and hyposmia since she was 66 years old. Her medical history was unremarkable. The patient visited the First People's Hospital of Jiande at the age of 68 when she was diagnosed with PD. She was then treated with benserazide/levodopa 12.5/50 mg three times daily, and her symptoms were almost completely relieved. Two years later, the drug was adjusted to benserazide/levodopa 25/100 mg three times daily and pramipexole 0.25mg three times daily as her symptoms had worsened. Her symptoms were still well controlled, and she did not suffer any motor fluctuation. However, the patient developed recurrent blepharospasm within the past 6 months. Her blepharospasm lasted approximately 30 min and was not complicated by worsened parkinsonism at the same time. To further investigate the relationship between blepharospasm and parkinsonism, a levodopa challenge test was performed. The patient took 50/200 mg of benserazide/levodopa, and we evaluated her at baseline, 15, 30, 45, and 60 min, and every 30 min thereafter up to 4 h. As shown in Figure 1, her parkinsonism was relieved within 30 min, and the improvement persisted until the end of the evaluation. Interestingly, she displayed blepharospasm for around 1 h, and it lasted for 30 min (Figures 2A,B; Supplementary Videos 1, 2). During this period, she developed mild dyskinesia in her left upper extremity, which was quickly relieved (within 10 min). Therefore, we diagnosed the blepharospasm as a manifestation of peak-dose dyskinesia. Because the patient refused a botulinum toxin A injection, we prescribed amantadine 100 mg two times daily. The drug worked within 2 days, with the best improvement within 2 weeks. The blepharospasm continued to improve over a follow-up period of 6 months. At present, the patient only has increased blinking after taking benserazide/levodopa, which does not obstruct the field of vision, and she is satisfied with the treatment.

## Discussion

Blepharospasm is a type of focal dystonia that may be idiopathic or secondary to a neurological condition such as PSP, tardive dyskinesia, and PD (11). It is of great concern to patients and physicians as it obstructs the field of vision and reduces the quality of life. In this report, we present the case of a patient with PD who developed blepharospasm as a manifestation of peak-dose dyskinesia, and it was relieved by taking amantadine.

Blepharospasm is uncommon in PD. Two independent studies reported its incidence as 0.9% (eight out of 913) (13) and 3.26% (nine out of 276) (12), respectively. However, it was more prevalent in PSP (6/57 and 7/10, respectively). Therefore, it is sometimes labeled as a clue to differentiate PD from atypical PD.

In idiopathic PD, blepharospasm is more recognized as a rare presentation of off-period dystonia (4, 14). Studies have also suggested that idiopathic blepharospasm is a risk factor for developing PD (15, 16). However, this conclusion is still controversial (17). In our report, we described a rare phenomenon in which blepharospasm appears as the main symptom of peak-dose dyskinesia in a patient with PD. Although the phenomenon is rare, it has been noticed and reported by other researchers (18).

The mechanisms underlying blepharospasm are, as yet, unknown. Multiple regions including the thalamus, lower brainstem, basal ganglia, cerebellum, midbrain, and cortex may participate in its pathophysiology (19). A functional magnetic resonance imaging-based study showed that basal ganglia circuits and cerebello-cortical circuits are involved in the triggering and development of blepharospasm (20). Interestingly, the two circuits also play an important role in PD (21). In addition, some pathological changes in advanced PD, such as hypersensitivity of the striatal dopaminergic receptors and abnormal striato-cortical connectivity, may add to the complexity of the mechanisms (4, 22). We suggest that “on” period and “off” period blepharospasm may be due to different pathological mechanisms.

Botulinum toxin A is the first choice in treatment of blepharospasm (23). Most patients benefit from it, and the improvement persists for several months (24). Trihexyphenidyl and clonazepam have been proven effective in improving blepharospasm (25, 26), but their application is limited due to the possibility of cognitive decline and sedative side effects. In our case, the patient refused the injection of botulinum toxin A. Given that blepharospasm is a manifestation of peak-dose dyskinesia, we tried amantadine, which is recommended by The International Parkinson and Movement Disorder Society (MDS) for treatment of dyskinesia (27), and it turned out effective in our patient.

To the best of our knowledge, it is rare that blepharospasm appears as the main manifestation of peak-dose dyskinesia in patients with PD, and its benefit from amantadine has not been reported before. Our report adds to the understanding and treatment of blepharospasm in PD.

## Data availability statement

The original contributions presented in the study are included in the article/Supplementary material, further inquiries can be directed to the corresponding author/s.

## Ethics statement

The studies involving human participants were reviewed and approved by the Ethics Committee of the First People's Hospital of Jiande. The patients/participants provided their written informed consent to participate in this study. Written informed consent was obtained from the individual(s) for the publication of any potentially identifiable images or data included in this article.

## Author contributions

J-YW, Q-YF, and XZ examined the patient and carried out the treatment strategy. Q-YF, X-DZ, Z-DH, C-PC, S-SH, and S-GZ acquired and analyzed all the clinical data. Z-DH and J-YW reviewed the literature and drafted the manuscript. XZ and J-YW supervised the study. All authors contributed to the article and approved the submitted version.

## Funding

The study was supported in part by funding from the Wenzhou Municipal Science and Technology Bureau (Y2020065), the Fundamental Research Funds for Wenzhou Medical University (KYYW202030), and the Novel Technology Program of the Second Affiliated Hospital and Yuying Children's Hospital (2022014).

## Conflict of interest

The authors declare that the research was conducted in the absence of any commercial or financial relationships that could be construed as a potential conflict of interest.

## Publisher's note

All claims expressed in this article are solely those of the authors and do not necessarily represent those of their affiliated organizations, or those of the publisher, the editors and the reviewers. Any product that may be evaluated in this article, or claim that may be made by its manufacturer, is not guaranteed or endorsed by the publisher.
